# Circular RNA EPB41 expression predicts unfavorable prognoses in NSCLC by regulating miR-486-3p/eIF5A axis-mediated stemness

**DOI:** 10.1186/s12935-022-02618-7

**Published:** 2022-06-20

**Authors:** Mingming Jin, Xiyu Liu, Yue Wu, Yuqing Lou, Xue Li, Gang Huang

**Affiliations:** 1 Shanghai Key Laboratory of Molecular Imaging, Shanghai University of Medicine and Health Sciences, 279 Zhouzhu Road, Pudong New Area, Shanghai, 201318 People’s Republic of China; 2 Shanghai University of Traditional Chinese Medicine, Shanghai, 201203 People’s Republic of China; 3 Health School Attached to Shanghai University of Medicine and Health Sciences, Shanghai, 200237 People’s Republic of China

**Keywords:** Non-small cell lung cancer, Circ-EPB41, miR-486-3p, eIF5A, Cancer stem cells

## Abstract

Dysregulation of circular RNAs (circRNAs) has recently been found to play an important role in the progression and development of cancers such as non-small cell lung cancer (NSCLC). Yet the functions of many circRNAs in NSCLC remain unclear. In this study, the circRNA expression profiles in NSCLC tumor tissues and adjacent non-tumorous tissues were detected by high-throughput sequencing. Bioinformatics analyses, the dual-luciferase reporter system, fluorescence in situ hybridization (FISH) and miRNA/mRNA high-throughput sequencing were used to identify circ-EPB41 and its downstream target. The subcutaneous tumor/caudal vein transfer mouse model was used for tumor growth and invasion analysis. The results show that the circ-EPB41 was upregulated in NSCLC tissues and cell lines. Increased circ-EPB41 expression in NSCLC was significantly correlated with malignant characteristics, and positive to post-surgical overall survival of NSCLC patients. Reduced circ-EPB41 expression in NSCLC decreased cell proliferation and invasion in both in vitro and in vivo experiments. The miRNA/mRNA high-throughput sequencing suggested that downregulation of circ-EPB41 promoted microRNA (miR)-486-3p and suppressed eukaryotic translation initiation factor 5A (eIF5A) expression. Luciferase reporter experiments confirmed that miR-486-3p/eIF5A were downstream targets of circ-EPB41. In addition, we also found that downregulation of circ-EPB41 suppressed self-renewal and decreased expression of stemness markers SOX2, OCT-4, Nanog and CD133 by sponging miR-486-3p to enhance eIF5A expression. Taken togeter, these data revealed the important role of circ-EPB41 in regulating NSCLC cell invasion and proliferation by modifying miR-486-3p/eIF5A axis-mediated stemness. We believe our study provides a novel perspective regarding the role of circRNAs in NSCLC progression.

**Supplementary Information:**

The online version contains supplementary material available at 10.1186/s12935-022-02618-7.

## Background

Lung cancer (LC) is among the most prevalent cancers, and its prevalence is a major factor affecting cancer mortality worldwide. There were about 0.73 million newly diagnosed cases and 0.61 million deaths because of LC in 2015 in China [1, 2]. Based on the annual predictions of the American Cancer Society, we can expect about 1.762 million new cancer patients and 606,880 cancer deaths in the US in 2019, of which LC will be among the most prevalent causes of both mortality and incidence [3]. Approximately 80–85% all human LCs are non-small cell lung cancer (NSCLC), consisting of two main subtypes, lung squamous cell carcinoma and lung adenocarcinoma [4, 5]. The 5-year relative survival rate is 57% for patients with stage I disease and declines to 4% for patients with stage IV disease [1]. Because of the lack of effective diagnostic methods and markers, the majority of NSCLC patients are diagnosed in the advanced stage, which is characterized by poor prognosis and distant metastasis [6]. Therefore, a better understanding of the molecular mechanisms that underlie NSCLC progression is indispensable for developing more effective therapies.

Circular RNAs (circRNAs) belong to a class of noncoding and protein-coding RNAs localized to the cytoplasm which originate from both single and multiple exons [7, 8]. Many studies have shown that circRNAs are involved in various pathological and physiological processes including cancer progression and tumorigenesis [9, 10]. For instance, circFGFR1 may interact directly with miR-381-3p and function as a miRNA sponge to upregulate expression of miR-381-3p target gene C-X-C motif chemokine receptor 4, which enhances NSCLC progression as well as resistance to anti-programmed cell death 1-based therapy [11]. Circ_0000003 promotes NSCLC cell metastasis and proliferation through miR-338-3p/insulin receptor substrate 2 [12]. Circ_0002483 has been verified to inhibit NSCLC progression in vivo and in vitro, and increases NSCLC cell sensitivity to Taxol through sponging of miR-182-5p to inhibit expression of FOXO1, FOXO3 and GRB2 mRNAs [13]. Our previous studies have found that circARHGAP10 suppresses NSCLC progression by functioning like a miR-150-5p sponge to enhance GLUT1 expression [14]. In NSCLC, previous investigations have discovered the existence of various circRNAs such as circ-ZKSCAN1, circ-CMPK1, circ-HIPK3 and circ-Foxo3 [15–18]. While the circRNA function and mechanism in NSCLC are not fully explored.

Current research has examined circRNA profiles expressed in human NSCLC tissues and shown that hsa_circ_0000042 (circ-EPB41) is a conserved circRNA, which is significantly upregulated in NSCLC tissues. In addition, circ-EPB41 expression is closely associated with NSCLC patient prognosis. We report here that circ-EPB41 promoted NSCLC progression by regulating miR-486-3p and eukaryotic translation initiation factor 5A (eIF5A)-mediated stemness. Thus, circ-EPB41 may serve as a novel marker for NSCLC prognosis and may be a promising therapeutic target.

## Materials and methods

### Tissue samples

In total, we collected 90 fresh NSCLC tissues with paired adjacent non-cancerous lung tissues after obtaining informed patient consent at Renji Hospital affiliated with Shanghai Jiaotong University, China. We evaluated pathological and histological diagnostics of NSCLC based upon the Revised International System for Staging Lung Cancer. Patients received no radiotherapy or chemotherapy prior to tissue sampling. We snap-froze samples in liquid nitrogen and stored them at − 80 °C before RNA extraction. The Ethics Committee of Shanghai University of Medicine and Health Sciences approved the study.

### Strand-specific RNA-Seq library construction and high-throughput RNA-Seq

We extracted total RNA from three paired NSCLC tissues as well as adjacent non-cancerous lung tissues using TRIzol Reagent (Invitrogen, Carlsbad, CA, USA). We subjected 3 μg of total RNA from each sample to the VAHTS Total RNA-Seq (H/M/R) Library Prep Kit from Illumina (Vazyme Biotech Co., Ltd, Nanjing, China) to remove ribosomal RNA and different classes of RNA such as non-coding RNA and mRNA. We treated purified RNA with RNase R (Epicentre Technologies, Madison, WI, USA; 40 U, 37 °C, 3 h), followed by TRIzol purification. We prepared RNA-Seq libraries using the KAPA Stranded RNA-Seq Library Prep Kit (Roche, Basel, Switzerland) and subjected them to deep sequencing with the Illumina HiSeq 4000 at Aksomics, Inc. (Shanghai, China; accession code: H1712024).

For miRNA and mRNA analysis, A549 cells transfected with siRNA against circ-EPB41 or a negative control (NC) vector were used for high-throughput RNA-Seq as previously described (accession code: H1712024).

### Cell lines and culture

We obtained the human normal lung epithelial cell line BEAS-2B and the NSCLC cell lines PC9, A549, H1975 and H1650 from the Cell Bank of the Chinese Academy of Sciences (Shanghai, China) and cultivated them in Dulbecco’s Modified Eagle’s Medium (DMEM; Life Technologies, Carlsbad, CA, USA) supplemented with 100 IU/mL penicillin, 100 μg/mL streptomycin and 10% fetal bovine serum (FBS; Invitrogen) at 37 °C in a humidified atmosphere with 5% CO_2_.

### Fluorescence in situ hybridization (FISH)

We obtained specific probes for circ-EPB41 (Dig-5ʹ-CATACTGCTCTTGCTCCATCGAGGCTCC-3ʹ-Dig) and miR-486-3p (Dig-5ʹ-CGGGGCAGCUCAGUACAGGAUA-3ʹ-Dig) from Geneseed Biotech (Guangzhou, China). Cells were immunostained with Cy3-conjugated anti-digoxin and FITC-conjugated anti-biotin antibodies (Jackson ImmunoResearch Inc., West Grove, PA, USA). We counterstained nuclei with 4,6-diamidino-2-phenylindole (DAPI). Staining was visualized with a Zeiss LSM 700 confocal microscope (Carl Zeiss, Oberkochen, Germany).

### Bioinformatics analyses

We imputed circRNA/miRNA target genes through the Interactome (https://circinteractome.nia.nih.gov/), miRanda (http://www.microrna.org/microrna/home.domiRanda) and circBank (http://www.circbank.cn/) websites. We calculated interactive correlations between miR-486-3p and eIF5A via the Targetscan website.

### Cell cycle detection

We dissociated cells in the logarithmic growth phase with 0.25% trypsin (Invitrogen, Carlsbad, CA, USA), resuspended them in PBS and fixed them in 70% ice-cold ethyl alcohol overnight at 4 °C. Afterwards, we centrifuged cells at 1200*g* for 5 min, resuspended them in 50 μL RNase A (Invitrogen, Carlsbad, CA, USA) and incubated them at 37 °C for 30 min. Then, we added 400 μL propidium iodide to the suspension for 30 min, followed by flow cytometric detection (BD Biosciences, San Jose, CA, USA).

### Plasmid construction and stable transfection

We synthesized human eIF5A and circ-EPB41 cDNA and then cloned it into the luciferase-labelled pcDNA3.1 vector (Invitrogen). We synthesized wild-type (WT) and mutant (MUT) circ-EPB41 and eIF5A cDNAs and cloned them into pZW1 vectors (Shanghai Institutes for Biological Sciences, Shanghai, China). We also transfected H1650 and A549 cells with the aforementioned plasmids.

To further validate the effects of circ-EPB41 in the in vivo experiments, a lentiviral-based small hairpin RNA (shRNA) (lentiviral vector: CMV-MCS-EF1α-GFP-PURO; Novagen) targeting circ-EPB41 was constructed. GFP detection was performed 72 h after infection into A549, and at a green fluorescence > 95%, the transfection was considered successful. For in vivo metastatic detection, pLVX-Luc2-P2A-AcGFP1-puro lentiviral vector (Novagen) was used for A549 infection and it generated the luc-A549 cell line.

### Total RNA isolation and the quantitative reverse transcription-polymerase chain reaction (RT-qPCR)

We isolated total RNA from tumor tissues and cells with TRIzol reagent following standard procedures. We examined the concentration and purity of RNA samples spectrophotometrically by capturing the absorbance at 230 nm, 260 nm and 280 nm with a NanoDrop ND-1000 (Thermo Fisher Scientific, Wilmington, DE, USA). We deemed OD260/OD280 ratios between 1.8 and 2.1 as acceptable, and also deemed OD260/OD230 ratios > 1.8 as acceptable.

We reverse transcribed total RNA before RT-qPCR detection. We obtained primers specific for circ-EPB41, eIF5A and miR-486-3p from GenePharma (Shanghai, China). We performed RT-qPCR using an AB7300 thermo recycler (Applied Biosystems, Carlsbad, CA, USA) with the primers listed below and TaqMan Universal PCR Master Mix. We utilized *GAPDH* as a reference gene for circRNAs and mRNAs and U6 RNA as an internal control for miRNA expression level. We quantified gene expression via the 2^−ΔΔCt^ method. The primers utilized to assay *circ-EPB41* expression were forward, 5′-AGGATCCAAATTTCGATACAGTGGC-3′ and reverse, 5′-ATTTCTTAGCTGCTCGGTAACTGGG-3′. The primers for *miR-486-3p* were forward, 5′-GCGGGGCAGCTCAGTA-3′ and reverse, 5′-GTTGGCTCTGGTGCAGGGTCCGAGGTATTCGCACCAGAGCCAACATCCTG-3′. The *eIF5A* primers were forward, 5′-GAGATGCAGGGGCCTCAGCCACC-3′ and reverse, 5′-GTGATAGGTACCCATCCTGGATG-3′. *U6* primers were forward, 5′-CTCGCTTCGGCAGCACA-3′ and reverse, 5′-AACGCTTCACGAATTTGCGT-3′. The *GAPDH* primers were forward, 5′-GCACCGTCAAGGCTGAGAAC-3′ and reverse, 5′-GGATCTCGCTCCTGGAAGATG-3′.

### RNA interference and overexpression

The miR-486-3p inhibitors and siRNA against *circ-EPB41 (sicirc-EPB41)* were purchased from GenePharma. Transfections were performed following the supplier's protocol. In brief, we transferred cells to 6-well culture plates and transfected them using Lipofectamine 2000 (Invitrogen). To achieve eIF5A overexpression, we transfected the pCDNA3.0 vector described above. For xenograft experiments, a lentiviral-mediated *circ-EPB41*-silencing vector (sh-circ-EPB41) was transfected into A549 cells.

### Tumor sphere formation assay

We harvested A549 and H1650 cells and resuspended them as single cells in serum-free medium. After precise cell counting, we added 200 cells/well in 200 μL of serum-free medium to a 96-well plate, 10 wells per group. We changed medium every 2 days. We took images of five randomly selected regions in each group of wells with a camera-equipped microplate reader (Leica, Wetzlar, Germany). We calculated sphere percentage by the number of spheres/200.

### Cell proliferation assay

We employed Cell Counting Kit-8 assays (CCK-8; Gibco, Logan, Utah, USA) to quantify cellular proliferation. We seeded transfected cells into 96-well plates at a density of 5,000 cells/well in triplicate. We detected cell viability with the CCK-8 system at 0, 24, 48 and 72 h after seeding, following standard procedure.

For colony formation assays, we seeded transfected cells into 6-well plates at a density of 2000 cells/well and maintained them in DMEM containing 10% FBS for 10 days. We imaged colonies and counted them after fixing and staining them.

### Transwell assay

For invasion assays, we placed Transwell assay inserts (Millipore, Billerica, MA, USA) into 24-well plates. The membrane in the upper Transwell chamber was coated with Matrigel (BD Biosciences). We placed 500 μL of DMEM containing 10% FBS into the bottom chamber and seeded 10,000 cells in 200 μL serum-free DMEM in the upper chamber. After 1–2 days, we utilized methanol to fix cells on the membrane and stained them with Crystal Violet. Finally, we observed the cells via a microscope (Leica).

### Western blot assay

We extracted protein from tissues or cells with RIPA lysis buffer and performed western blot assays as previously described [19]. We obtained primary antibodies against SOX2, OCT-4, Nanog, CD133 and GAPDH from Cell Signaling Technology (Beverly, MA, USA) and stained protein blots following standard procedures. We visualized immunoreactivity with a chemiluminescence detection kit (Western Blotting Substrate, Donghuan Biotech, China).

### Immunofluorescence

We fixed cells or tumor tissues in 4% paraformaldehyde for 20 min at room temperature (RT). We then incubated the fixed cells or tissues with 0.5% Triton-X-100 in PBS for five min at RT and blocked them with 5% bovine serum albumin in PBS for 1 h at RT. The cells were incubated with primary antibodies against SOX2 (1:200; Abcam, Cambridge, MA, USA) at 4 °C overnight, followed by incubation with fluorochrome-conjugated secondary antibody (1:200; Abcam) and Nestin primary antibody conjugated to FITC (1:200; Millipore) at RT for 1 h. We stained nuclei in DAPI for 15 min at RT and visualized staining via fluorescence microscope.

### The 5-ethynyl-2′-deoxyuridine (EdU) assay

We purchased an EdU assay kit (RiboBio, China) to measure cell proliferation and DNA synthesis. We seeded 10,000 A549 or H1650 cells in 96-well plates overnight, then added Edu solution (25 μM) to the wells for 1 day. We applied 4% formalin to fix the cells at RT for 2 h and permeabilized the cells with 0.5% TritonX-100 for 10 min. We then added 200 μL of Apollo reaction solution for 30 min to stain EdU and 200 μL of DAPI to stain the nuclei. Lastly, we utilized a Nikon microscope (Nikon, Japan) to detect cell proliferation and DNA synthesis, reflected by blue and red signals, respectively.

### Dual-luciferase reporter assay

We constructed WT/mut-circ-EPB41 or WT/mut-eIF5A 3'UTR fragments and inserted them downstream into a pMIR-REPORT plasmid luciferase reporter gene (H306; Obio Technology, Shanghai, China). We employed Lipofectamine 2000 to transfect the reporter plasmid into A549 cells and co-transfected the miR-486-3p mimic with the reporter gene into HEK293T cells. Afterwards, we employed the Dual Luciferase Reporter System Kit (E1910; Promega, Madison, WI, USA) to measure firefly and Renilla luciferase activity.

### Animal studies

To detect the role of circ-EPB41 in a LC metastasis model, we intravenously injected 1 × 10^6^ stable lentiviral-mediated circ-EPB41-silenced A549 cells (sh-circ-EPB41) or A549 NC cells into male nude mice (Chinese Science Academy, Shanghai, China) via the tail vein. After 1 month, we analyzed A549 cell metastasis by bioluminescence imaging following an intravenous luciferin injection (150 mg luciferin/kg body weight) into the tails. The numbers of metastatic foci in lung tissues were caculation according to the HE staining.

For xenograft assays, we injected 1 × 10^6^ modified (circ-EPB41) or control (WT) A549 cells subcutaneously into the right flank of male nude mice. We calculated tumor volumes (length × width^2^ × 0.5) at the indicated time points and excised the tumors 4 weeks after injection.

The mice were killed by cervical dislocation and the tumors were collected. The animal protocols complied with the rule of the ethics committee of Shanghai University of Medicine and Health Sciences.

### Immunohistochemistry

We fixed tumor tissue samples in 10% formalin and embedded them in paraffin. We stained sections (5-μm thick) with Ki67 to evaluate proliferation. We examined sections with an Axiophot light microscope and imaged them by digital camera.

### Statistical analyses

We assessed differences between any two groups by paired/unpaired two-tailed *t*-tests. We used Pearson’s correlation test to detect correlations between groups. We expressed data as means ± SD. A P-value < 0.05 was regarded as significant. We performed statistical analysis using the GraphPad Prism package (GraphPad Inc., San Diego, CA, USA).

## Results

### High circ-EPB41 expression in NSCLC is correlated with poor prognosis.

**Fig. 1:**
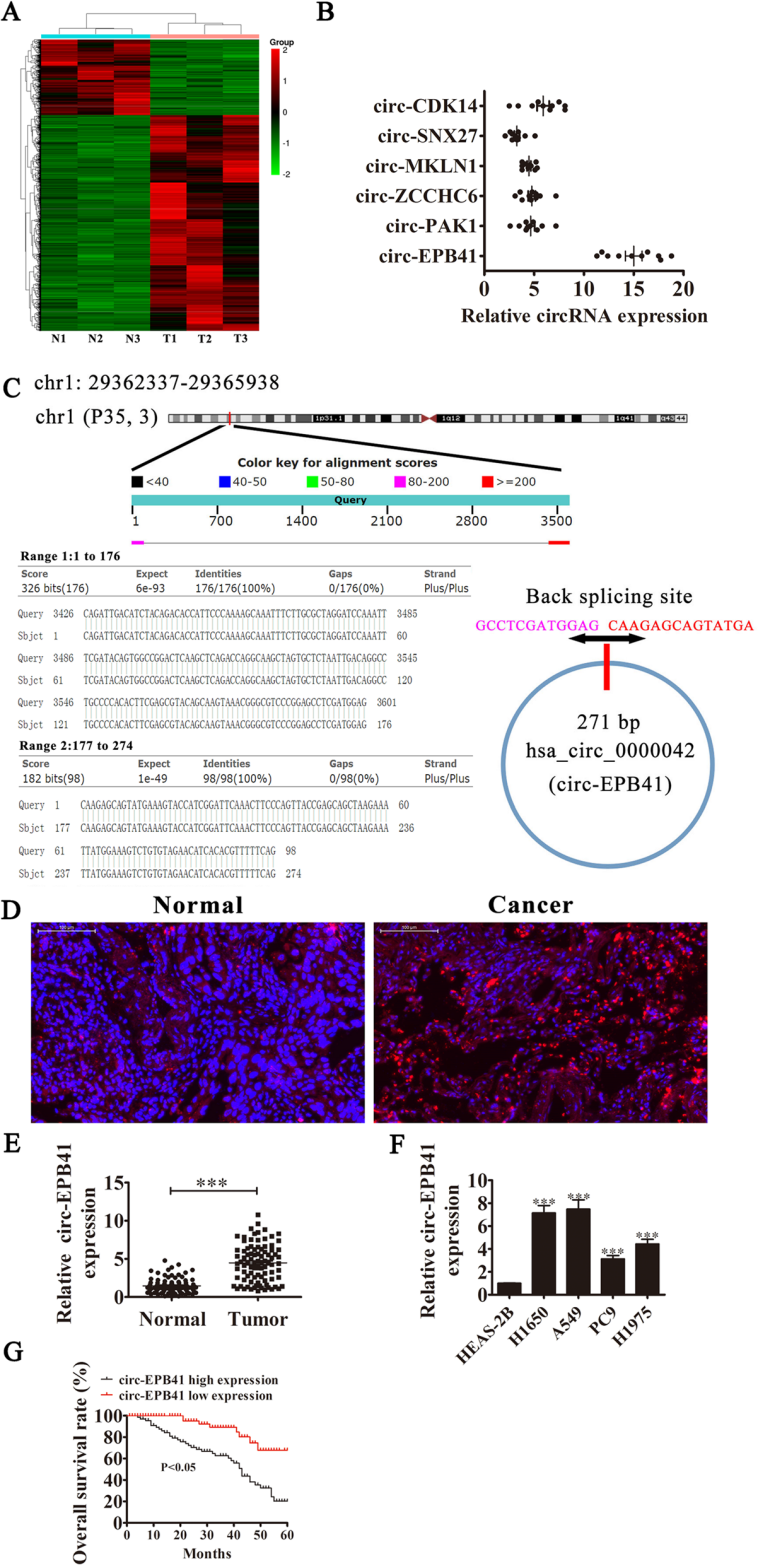
The expression of circ-EPB41 predicted an unfavorable prognosis in NSCLC patients. **A** Clustered heat map for tissue-specific circRNAs from three normal and cancerous human tissues. **B** We validated the differential expression of six circRNAs in 10 paired NSCLC tissues using RT-qPCR. **C** The genomic loci of the *EPB41* gene and circ-EPB41. **D** The expression and subcellular localization of circ-EPB41 in NSCLC was analyzed by in situ hybridization on a NSCLC tissue. **E** RT-qPCR detection showed the expression of circ-EPB41 in 90 paired NSCLC tissues. Data are presented as means ± SD; ^***^P < 0.001 vs normal group. **F** RT-qPCR detection showed the expression of circ-EPB4 in A549, PC9, H1650, H1975 and normal lung epithelial cells (BEAS-2B). Data are presented as means ± SD; ^***^P < 0.001 vs normal group. **G** The overall survival probability of patients with higher and lower circ-EPB41 were determined by Kaplan–Meier survival analysis

To verify the correlation between abnormal circRNA expression and NSCLC progression, we performed RNA-seq analyses on ribosomal RNA-depleted total RNA from three NSCLC and three adjacent normal tissue samples. The data indicated that 22 circRNAs were upregulated and 23 circRNAs were downregulated (Additional file 1; Fig. 1A). To validate the RNA-seq results, we selected six upregulated circRNAs (hsa_circ_0001721 [circ-CDK14], hsa_circ_0000130 [SNX27], hsa_circ_0001747 [circ-MKLN1], hsa_circ_0001869 [circ-ZCCHC6], hsa_circ_0008992 [circ-PAK1] and hsa_circ_0000042 [circ-EPB41]) for RT-qPCR analysis using ten paired NSCLC tissues. Results were consistent with RNA-seq data and we also found that circ-EPB41 expression was significantly enhanced in NSCLC tissues comparing with non-tumorous tissues (increased 15 times, the median value was 3.5; Fig. 1B).

Among these specific candidates, circ-EPB41 with higher expression levels was examined in more detail. We found that circ-EPB41 originated by circularization of two exons from the *EPB41* gene, which is located at chr1:29362337-29365938. *EPB41* consists of 3601 bp and the spliced mature circRNA is 271 bp (Fig. 1C). In order to further identify the subcellular localization of circ-EPB41, specific probes for circ-EPB41 were constructed across the back-splicing site. FISH assays detected that circ-EPB41 was localized mainly to the cytoplasm, and circ-EPB41 expression in NSCLC tissues was significantly increased compared to matched non-tumorous tissues (Fig. 1D). RT-qPCR detection also showed that circ-EPB41 expression was increased in NSCLC tissues using 90 paired NSCLC tissues (Fig. 1E). Furthermore, RT-qPCR detection showed that the expression of circ-EPB4 in A549, PC9, H1650 and H1975 cells was increased compared to normal lung epithelial cells (BEAS-2B; Fig. 1F). A549 and H1650 cells had the highest expression of circ-EPB41 and were selected for further study. Both RT-qPCR and FISH assays confirmed that circ-EPB41 was a circular RNA, because both promer and FISH probes were constructed across the back-splicing site.

We divided 90 paired NSCLC tissues into relatively high-expressing (n = 46) and relatively low-expressing (n = 44) tissues compared to normal circ-EPB41 expression. There was no correlation between circ-EPB41 expression and demographic factors such as sex or patient age (≤ 60 years vs > 60 years). However, high expression of circ-EPB41 was positively related to lymph node metastasis (negative vs positive), TNM stage (I/II or III/IV vs high) and tumor size (≤ 3 cm vs > 3 cm; Table 1). Also, Gehan-Breslow-Wilcoxon test survival curves demonstrated that NSCLC patients with high circ-EPB41 expression exhibited poor overall survival (Fig. 1G). These data suggested that the circ-EPB41 expression has an important function in NSCLC progression.

**Table 1:** The clinic-pathological factors of 90 NSCLC patients

Characteristics	Numbers	circ-EPB41		*P* value
		Low (N = 44)	High (N = 46)	
Sex				0.766
Male	48	26	22	
Female	42	18	24	
Age				0.883
≤ 50	43	23	20	
> 50	47	21	26	
TNM stage				0.024
I and II	52	35	17	
III and IV	38	9	29	
Lymph node metastasis				0.015
Negative	41	29	12	
Positive	49	15	34	
Tumor size				0.011
≤ 3 cm	48	34	14	
> 3 cm	42	10	32	

### The circ-EPB41 downregulation suppresses NSCLC invasion and proliferation in vivo and in vitro

**Fig. 2:**
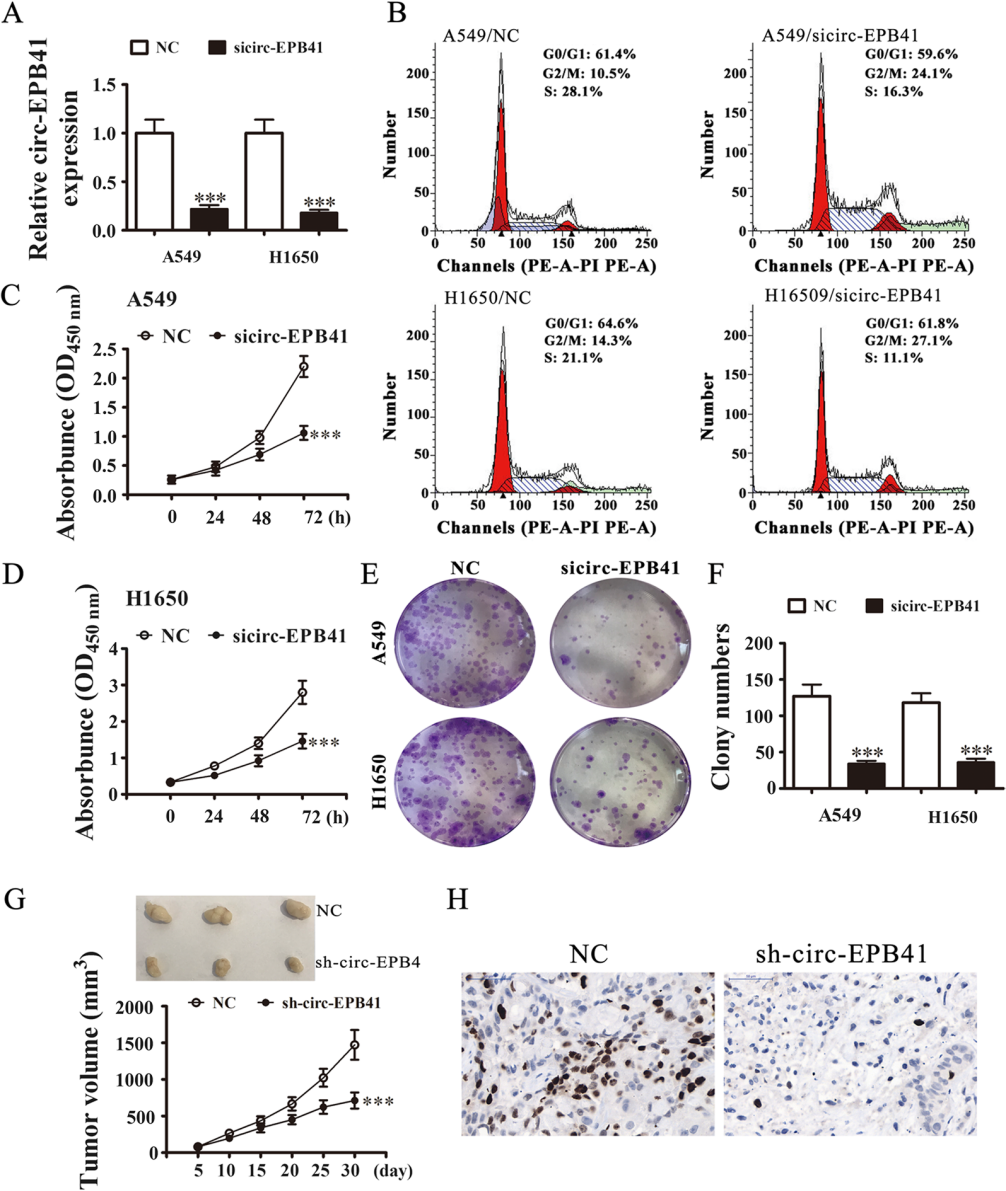
Downregulation of circ-EPB41 suppressed NSCLC proliferation both in vitro and in vivo. **A** RT-qPCR detection showing the expression of circ-EPB41 in both A549 and H1650 cells after transfection with sicirc-EPB41 or NC. Data are presented as means ± SD; ^***^P < 0.001 vs NC. **B** Flow cytometry detection showing the percentages of cells in G1, S or G2 phase for both A549 and H1650 cells. **C**, **D** CCK8 assays were used to evaluate cell proliferation in both A549 (**C**) and H1650 (**D**) cells. Data are presented as means ± SD; ^***^P < 0.001 vs NC. **E**, **F** Colony formation assays showing proliferation of both A549 and H1650 cells. Data are presented as means ± SD; ^***^P < 0.001 vs NC. **G** In xenograft tumor studies, A549 cells transfected with NC or sh-circ-EPB41 were subcutaneously injected into nude mice and tumor growth curves were plotted. Data are presented as means ± SD; ^***^P < 0.001 vs NC. (H) Immunohistochemistry showing the percentage of Ki67-positive cells. The relative levels Ki67-positive cells were calculated. Data are presented as means ± SD; ^***^P < 0.001 vs NC

To identify circ-EPB41’s role in NSCLC proliferation, we used H1650 and A549 cells. RT-qPCR analyses suggested that circ-EPB41 expression significantly decreased after transfection with sicirc-EPB41 in H1650 and A549 cells compared with the NC group (Fig. 2A). Cell cycle analysis by flow cytometry conducted on A549 cells illustrated that circ-EPB41 knockdown resulted in mild cell cycle arrest at the G0/G1 phase in nearly 14% of cells (Fig. 2B). CCK8 detection (Fig. 2C, D) and colony formation assays (Fig. 2E, F) demonstrated that circ-EPB41 silencing suppressed cell proliferation of H1650 and A549 cells. We then examined the effect of circ-EPB41 knockdown on tumor formation in a stable lentiviral strain (sh-circEPB41) or in sh-NC A549 cells. Xenograft results confirmed that circ-EPB4 knockdown suppressed tumor growth, as determined by tumor volume and immunohistochemistry (Fig. 2G) using Ki67 staining (Fig. 2H), compared with the NC group.

**Fig. 3:**
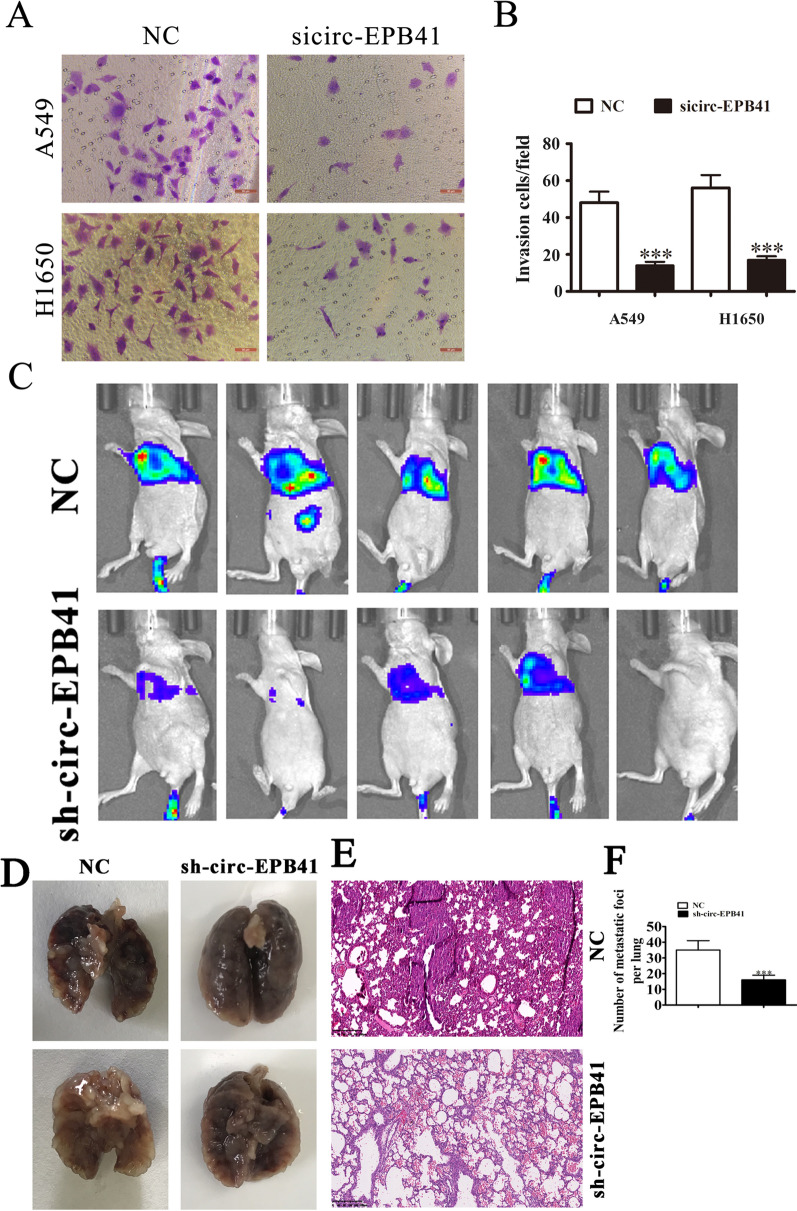
Downregulation of circ-EPB41 suppressed NSCLC invasion both in vitro and in vivo. **A**, **B** Cell invasion was assessed in both A549 and H1650 cells using Transwell assays. Data are presented as means ± SD; ^***^P < 0.001 vs NC. **C** Live imaging showing the effects of circ-EPB41 on the metastasis of A549 cells 30 days after intravenous tail injection in different groups. **D**–**F** The numbers of metastatic foci in lung tissues were caculation according to the HE staining. The data are expressed as the mean ± SD. ^***^p < 0.001 vs NC

Transwell assays demonstrated that circ-EPB41 downregulation suppressed NSCLC invasion in H1650 and A549 cells (Fig. 3A, B). We also employed a small animal live imaging system to obtain fluorescence images from nude mice 30 days after tail-vein inoculation of circ-EPB41-silenced and control cells (Fig. 3C). The fluorescence intensity in the circ-EPB41-silenced group was consistently weaker than the NC group. HE staining analysis show that circ-EPB41 silence decreased the pulmonary metastasis ability by decreased the numbers of metastatic foci in lung tissues after HE staining analysis (Fig. 3D–F).

### The miR-486-3p and eIF5A are downstream targets of circ-EPB41

**Fig. 4:**
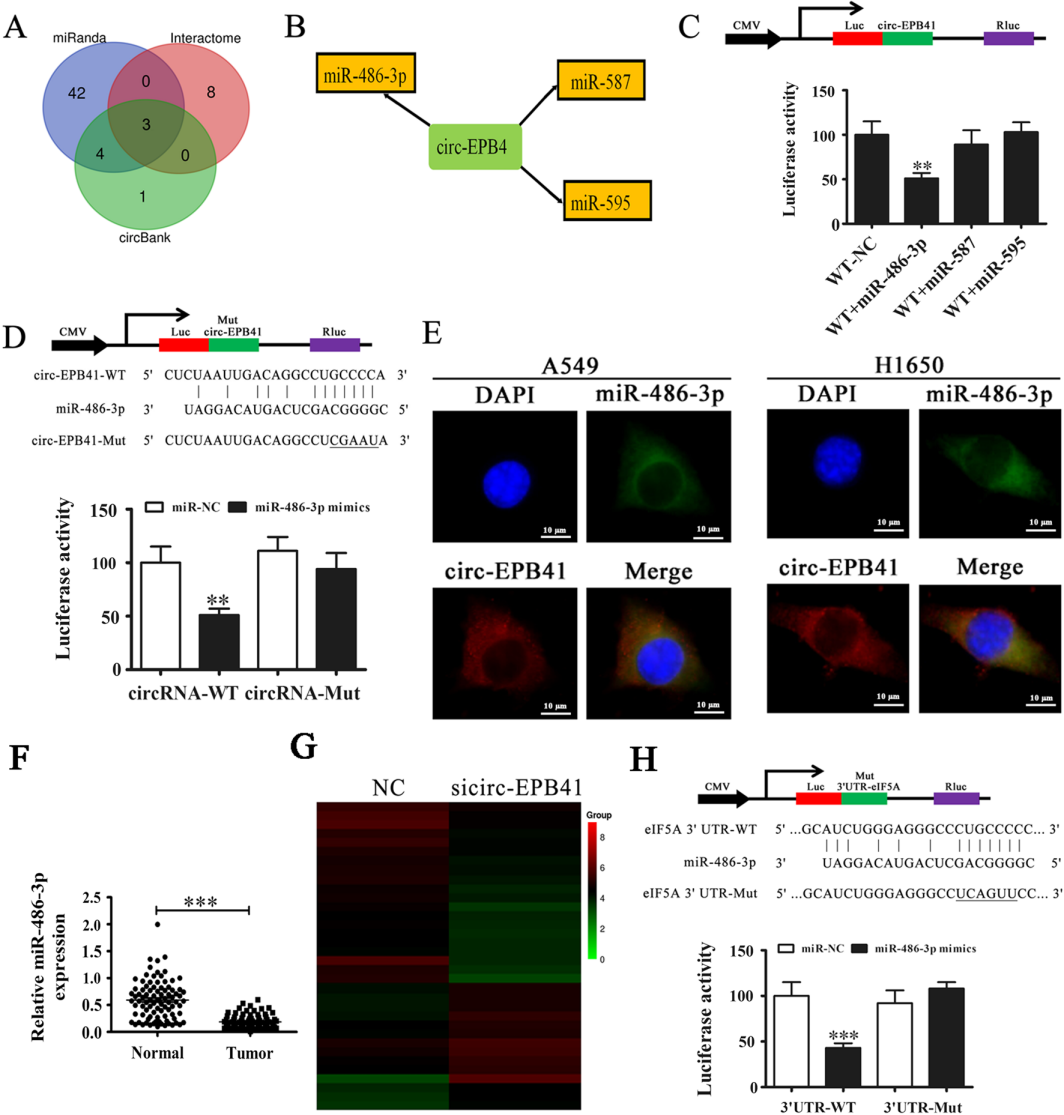
miR-486-3p and eIF5A are downstream targets of circ-EPB41. **A** Venn diagrams from the Interactome, miRanda and circBank websites for predicting miRNAs sponged by circ-EPB41. **B** Bioinformatics analysis (http://circnet.mbc.nctu.edu.tw/) found that miR-486-3p, miR-587 and miR-595 were targets of circ-EPB41. **C** Dual-luciferase reporter assays showed that co-transfection of WT and the mimic miR-486-3p markedly decreased luciferase activity in HEK293T cells. **D** Results of dual-luciferase reporter assays indicated that luciferase activity did not change in HEK293T cells when miR-486-3p binding sites in circ-EPB41 were mutated. Data are presented as means ± SD; ^**^P < 0.01. **E** Fluorescence in situ hybridization found that miR-486-3p co-localized with circ-EPB41 in the cytoplasm. **F** RT-qPCR detection showed the expression of miR-486–3 in 90 paired NSCLC tissues. Data are presented as means ± SD; ^***^P < 0.001 vs normal group. **G** Clustered heat map of differentially expressed mRNAs in A549 cells after downregulation of circ-EPB41. **H** Bioinformatics analysis (http://www.targetscan.org/vert_71/) found that eIF5A was the direct target of miR-486-3p. The MUT version of the 3ʹUTR-eIF5A is also shown. Relative luciferase activity was determined 48 h after transfection with miR-486-3p mimic/NC or with the 3'UTR-eIF5A WT/MUT in HEK293T cells. Data are presented as means ± SD; ^***^P < 0.001

Next, we investigated how circ-EPB41 regulation affects NSCLC proliferation and invasion. Previous studies have found that circRNA can act as a miRNA sponge. Given that circ-EPB41 is mainly localized to the cytoplasm, we hypothesized that circ-EPB41 might regulate tumor biological behavior by sponging miRNAs. We employed Interactome, miRanda and circBank to predict miRNAs targeted by circ-EPB41. The results showed that three miRNAs could be sponged by circ-EPB41 (Fig. 4A). Bioinformatics analysis further found that miR-587, miR-486-3p and miR-595 were circ-EPB41 targets (Fig. 4B; Additional file 2). High-throughput sequencing for miRNA expression using A549 cells found that downregulation of circ-EPB41 promoted miR-486-3p expression (Additional file 3). To verify this finding, we constructed a dual-luciferase reporter system by inserting the circ-EPB41 sequence into the psiCHECK2 plasmid (WT). The data showed that when co-transfected with WT or NC and miRNAs, only mimic miR-486-3p significantly decreased luciferase activity (Fig. 4C), suggesting that miR-486-3p was the direct target of circ-EPB41. We then cloned circ-EPB41 sequences containing mutated miR-486-3p binding sites into the psiCHECK2 plasmid and observed no obvious alterations in luciferase activity after co-transfection with MUT and corresponding miR-486-3p mimic (Fig. 4D). FISH detection showed that circ-EPB41 and miR-486-3p interacted and were co-localized in the cytoplasm of both A549 and H1650 cells (Fig. 4E). RT-qPCR detection also found that miR-486-3p expression was increased NSCLC cancer tissues when compared with tumor-adjacent tissue (Fig. 4F). High-throughput sequencing of A549 mRNA expression found that downregulation of circ-EPB41 decreased eIF5A expression (Fig. 4G). To verify that eIF5A was a miR-486-3p target, WT and MUT eIF5A 3'UTR sequences were cloned into the psiCHECK2 plasmid. The data revealed that while miR-486-3p mimic and reporter plasmid co-transfection visibly suppressed luciferase activity, miR-486-3p mimic and mutated eIF5A 3'UTR vector co-transfection did not have a significant effect on luciferase activity (Fig. 4H). Therefore, the data demonstrated that miR-486-3p directly targeted eIF5A.

### The circ-EPB41 promotes NSCLC progression by regulating miR-486-3p/eIF5A axis-mediated stemness

**Fig. 5:**
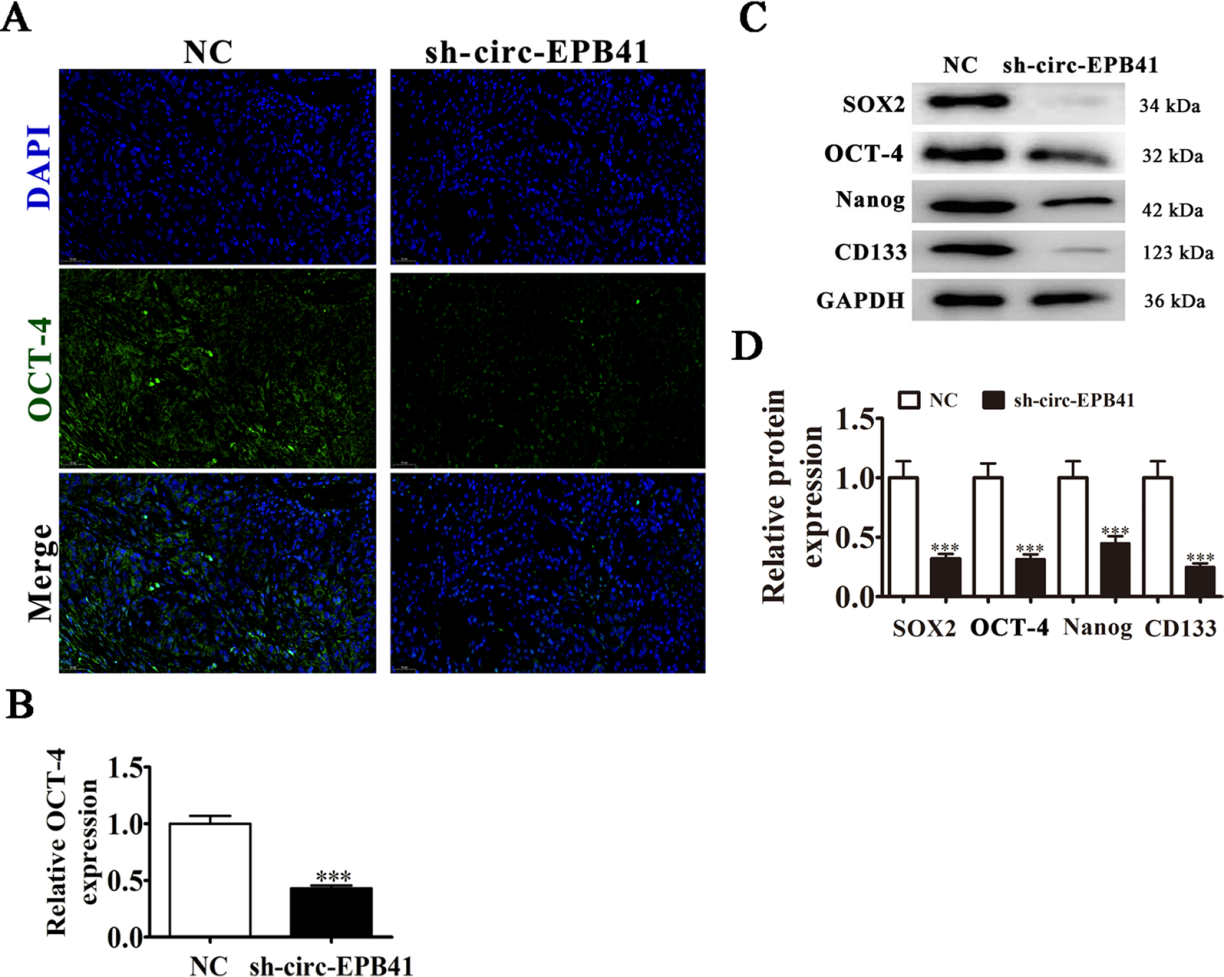
circ-EPB41 promoted the progression of NSCLC by regulating miR-486-3p/eIF5A axis-mediated stemness. **A**, **B** Immunofluorescence detection of stemness marker OCT-4 in tumor tissues. Data are presented as means ± SD; ^***^P < 0.001 vs NC. **C**, **D** Western blot detection shows the expression of stemness markers SOX2, OCT-4, Nanog and CD133 in tumor tissue with and without silencing of circ-EPB41. Data are presented as means ± SD; ^***^P < 0.001 vs NC

Previous studies have found that eIF5A can regulate cancer stem cells (CSCs) [20–23]. In the present study we examined if circ-EPB41 can regulate CSCs in NSCLC. Immunofluorescence detection of OCT-4 staining found that circ-EPB41 silencing suppressed stemness marker OCT-4 expression in tumor tissues (Fig. 5A, B). Western blot detection also found that expression of stemness markers SOX2, OCT-4, Nanog and CD133 was decreased after circ-EPB41 silencing in tumor tissues (Fig. 5C, D), suggesting that circ-EPB41 promoted the progression of NSCLC by regulating stemness.

**Fig. 6:**
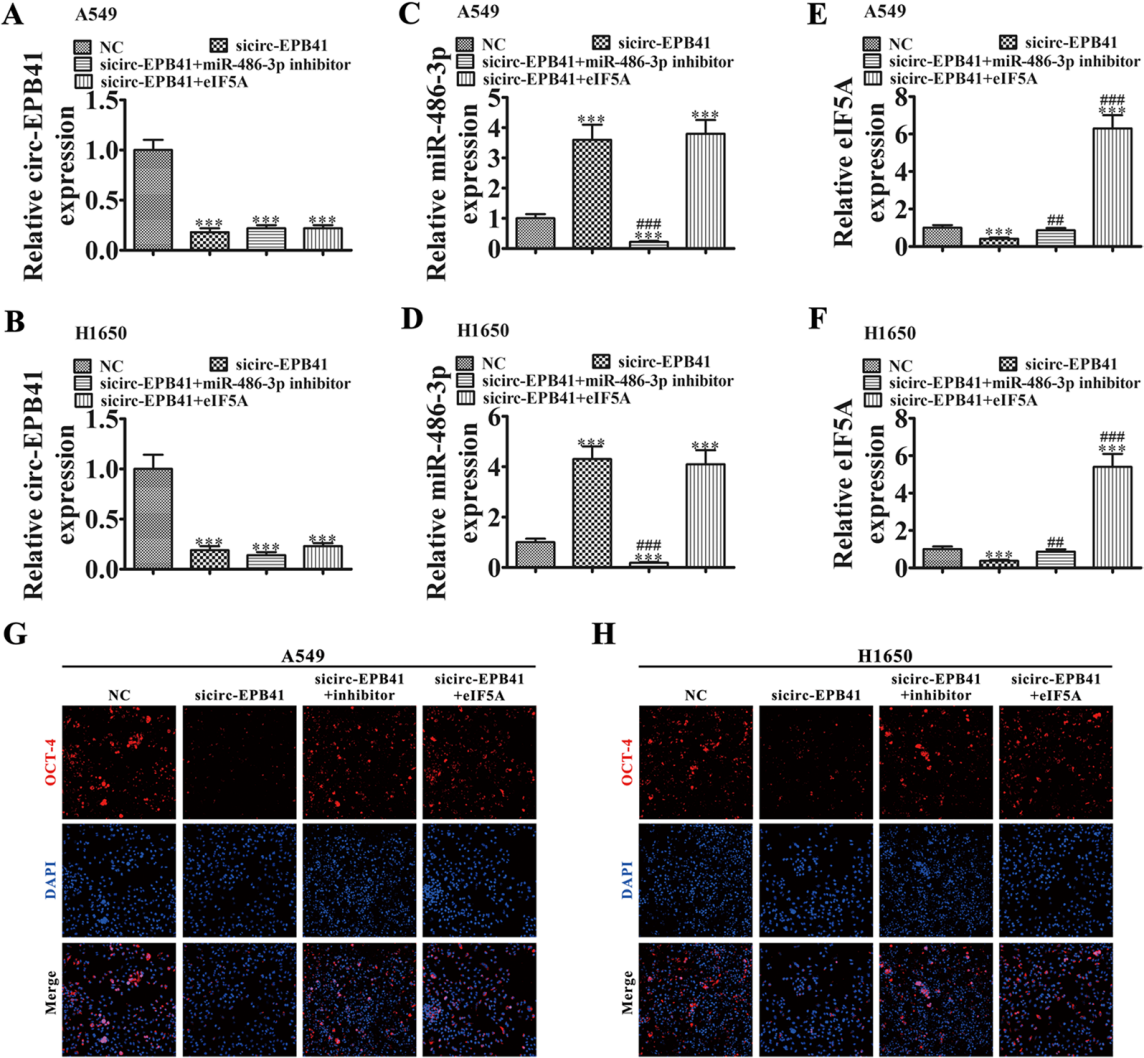
Downregulation of circ-EPB41 suppressed tumor stemness by regulating the miR-486-3p/eIF5A axis. **A**–**C** RT-qPCR detection shows the expression of circ-EPB41 (**A**–**F**), miR-486-3p (**C**, **D**) and eIF5A. **E**, **F** in both A549 and H1650 cells after transfection with NC, sicirc-EPB41, miR-486-3p inhibitor and eIF5A overexpression vector singly or combined. Data are presented as means ± SD; ^***^P < 0.001 vs NC; ^###^P < 0.001, ^##^P < 0.01 vs sicirc-EPB41. **G**, **H** Immunofluorescence of OCT-4 in both A549 and H1650 cells

In order to identify the interaction among circ-EPB41, miR-486-3p and eIF5A that regulates stemness, we transfected H1650 and A549 cells with negative vector (NC), sicirc-EPB41, miR-486-3p inhibitor and eIF5A overexpression vector singly or in combination. RT-qPCR detection showed that downregulation of circ-EPB41 suppressed circ-EPB41 expression, while downregulation of miR-486-3p or overexpression of eIF5A could not restore circ-EPB41 expression after circ-EPB41 silencing in A549 and H1650 cells (Fig. 6A, B). The results also found that downregulation of circ-EPB41 promoted miR-486-3p expression, but after treatment with miR-486-3p inhibitor, miR-486-3p expression was suppressed in A549 and H1650 cells. Upregulation of eIF5A could not suppress miR-486-3p expression after circ-EPB41 was silenced (Fig. 6C, D). RT-qPCR detection of eIF5A indicated that circ-EPB41 silencing suppressed eIF5A expression. Meanwhile, downregulation of miR-486-3p restored eIF5A expression, and eIF5A overexpression increased eIF5A expression in H1650 and A549 cells (Fig. 6E, F).

**Fig. 7:**
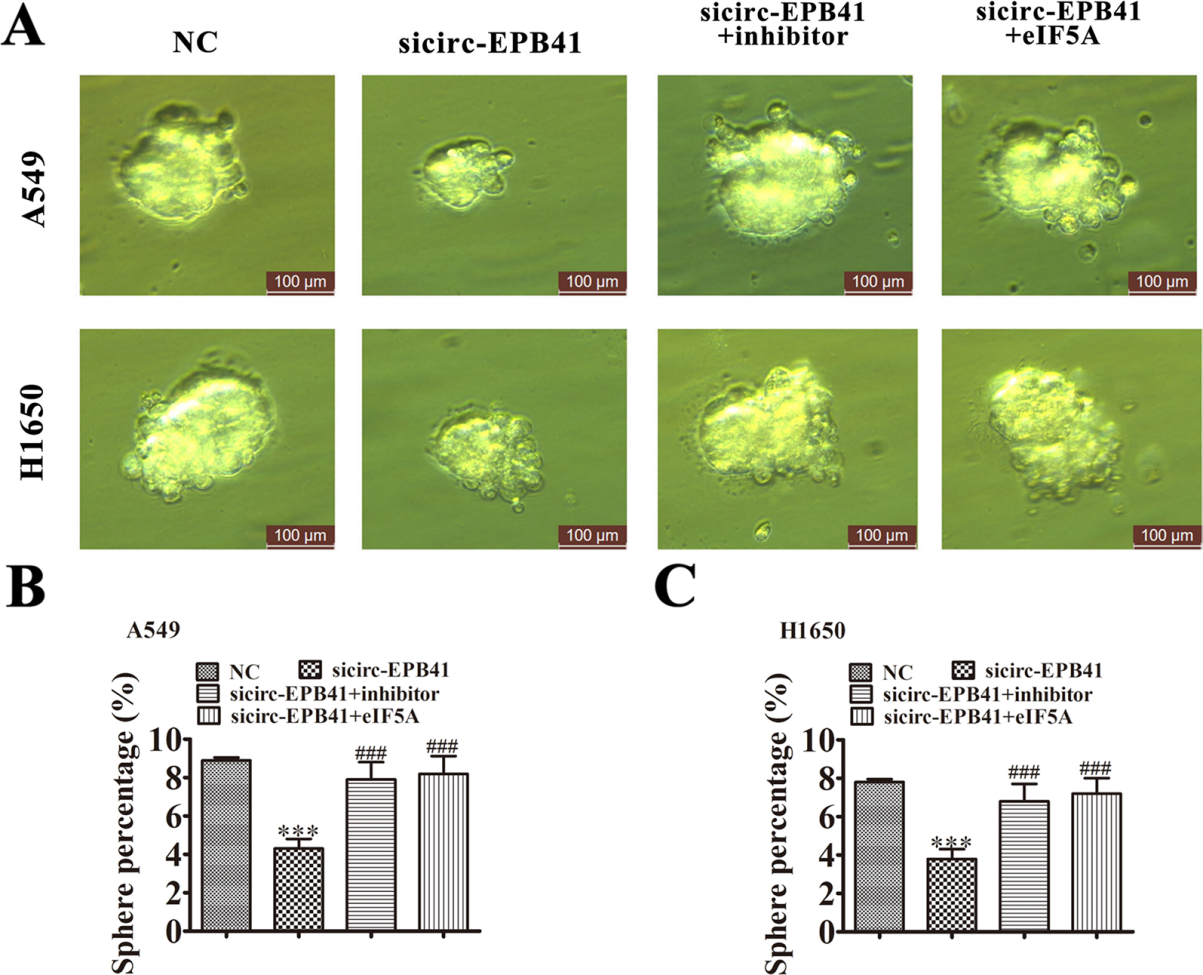
Downregulation of circ-EPB41 suppressed tumor sphere formation by regulating the miR-486-3p/eIF5A axis. **A** Images of tumor sphere formation assays in both A549 and H1650 cells (200 cells/well); scale bar, 100 μm. **B**, **C** Analyses of tumor sphere formation results indicated that downregulation of circ-EPB41 significantly inhibited self-renewal of NSCLC cells, whereas downregulation of miR-486-3p or overexpression of eIF5A restored self-renewal capacity. n = 10. Data are presented as means ± SD; ^***^P < 0.001 vs NC, ^###^P < 0.001 vs sicirc-EPB41

Immunofluorescence detection with OCT-4 staining found that silencing of circ-EPB41 suppressed OCT-4 expression in both A549 and H1650 cells, while downregulation of miR-486-3p or overexpression eIF5A restored stemness relative protein OCT-4 expression after circ-EPB41 silencing (Fig. 6G, H). We then conducted tumor sphere formation assays. These results indicated that downregulation of miR-486-3p or overexpression of eIF5A restored the sphere percentage in H1650 and A549 cells (Fig. 7A–C), suggesting that circ-EPB41 expression enhanced NSCLC progression by regulating miR-486-3p/eIF5A axis-mediated stemness.

### Downregulating miR-486-3p or overexpressing eIF5A restores cell proliferation and invasion after circ-EPB41 silencing

**Fig. 8:**
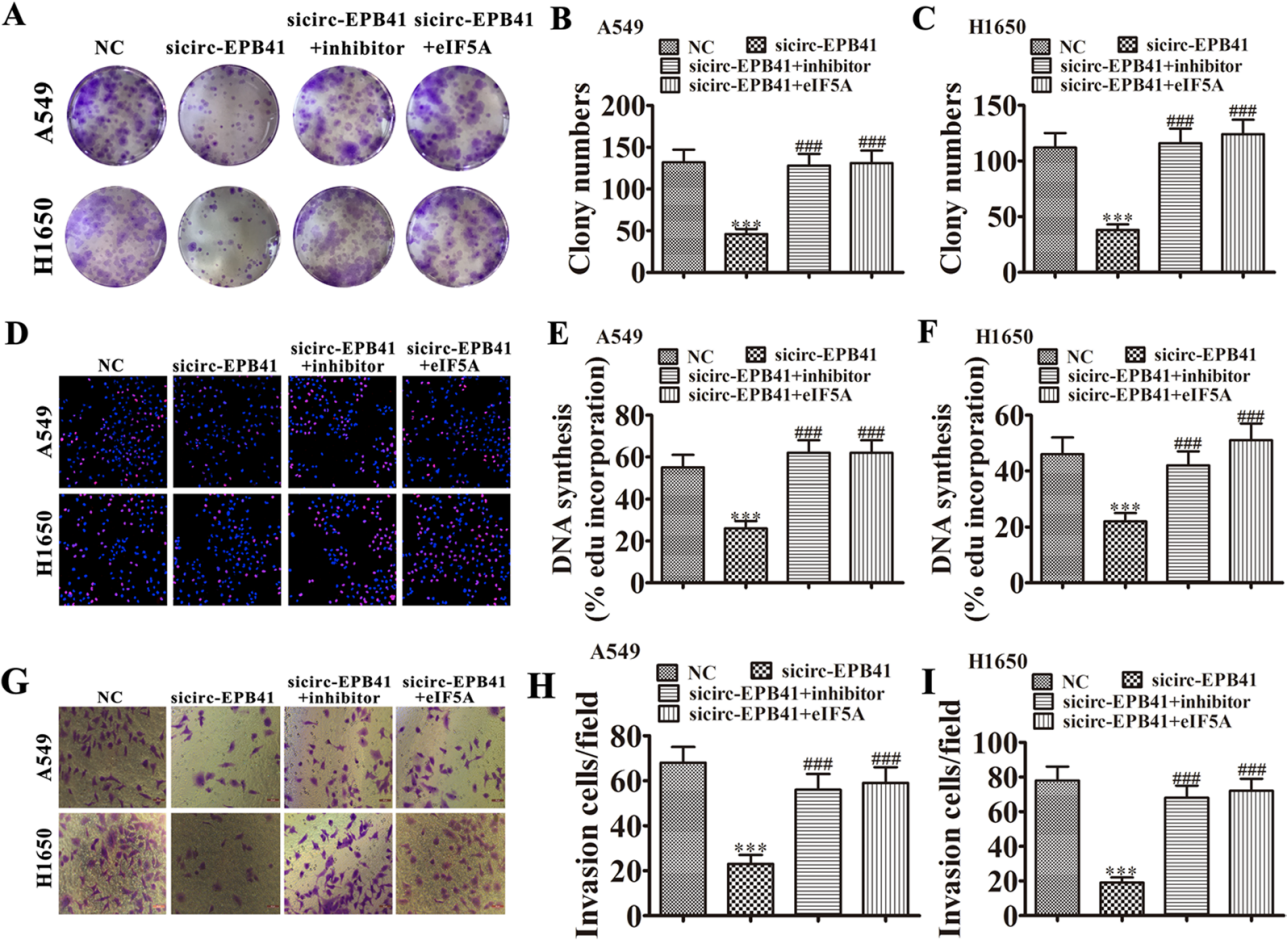
Downregulation of miR-486-3p or overexpression of eIF5A restored cell proliferation and invasion after circ-EPB41was silenced. **A**–**C** Cell proliferation was analyzed by colony formation (**A**–**C**) and Edu incorporation assays (**D**–**F**). Data are presented as means ± SD; ^***^P < 0.001 vs NC, ^###^P < 0.001 vs sicirc-EPB41. **G**–**I** Cell invasion was assessed in both A549 and H1650 cells using Transwell assays. Data are presented as means ± SD; ^***^P < 0.001 vs NC, ^###^P < 0.001 vs sicirc-EPB41

Our study also found using colony formation assays and Edu assays in H1650 and A549 cells that downregulation of miR-486-3p or overexpression of eIF5A renewed proliferative ability after eIF5A was silenced (Fig. 8A–F). Transwell invasion analysis also demonstrated that downregulating miR-486-3p or overexpressing eIF5A restored invasive ability after eIF5A was silenced in A549 and H1650 cells (Fig. 8G–I).

## Discussion

During last few decades, various studies have discovered abnormal non-coding RNA expression profiles in a number of cancers. Investigations have determined that many miRNAs and circRNAs have vital functions in modulating cancer metastasis and tumor growth [24, 25], although the mechanisms by which circRNAs participate in cancer progression and development are still unknown [26, 27]. To date, a few circRNAs have been shown to act critically in NSCLC [11, 13, 28–30]. Here, we report that circ-EPB41 is an essential circRNA that is frequently upregulated in NSCLC tissues, and that high expression predicts poor prognosis for lymph node metastasis, high TNM stage and large tumor size. Downregulation of circ-EPB41 induced G2/M arrest in NSCLC cells, while circ-EPB41 silencing suppressed NSCLC proliferation and metastasis in in vitro and in vivo experiments, suggesting that circ-EPB41 functions in NSCLC progression.

Circ-EPB41 originates from two *EPB41* gene exons. FISH detection indicated that circ-EPB41 is localized to cytoplasm. Additional published evidence implies that circRNAs function as miRNA sponges or as endogenous RNA (ceRNA) competitors to decrease functional dysregulation of miRNAs and their target genes, causing tumor proliferation and invasion, including in NSCLC [31, 32]. In the present study, A549 cells with or without circ-EPB41 silencing were used for high-throughput sequencing analysis. The results showed that downregulating circ-EPB41 resulted in a series of abnormally expressed mRNAs and miRNAs. Bioinformatics analysis found that miR-486-3p, miR-587 and miR-595 were the circ-EPB41 targets. Luciferase reporter assays and FISH analysis further confirmed that miR-486-3p was the direct target of circ-EPB41. Previous studies have confirmed that miR-486-3p expression was decreased in tumor tissues of LC patients [33]. Increased miR-486-3p represses cells metastasis and proliferation in many tumors including cervical cancer [34], oral cancer [35] and squamous cell carcinoma [36], suggesting that circ-EPB41 overexpression also suppressed NSCLC cell metastasis and proliferation via adsorption of miR-486-3p.

Further study with high-throughput sequencing and bioinformatics analysis found that miR-486-3p could target the 3'UTR of eIF5A. Previous studies have also found that eIF5A overexpression was associated with poor survival in many tumors [37, 38]. In the current study, we discovered that downregulation of miR-486-3p or overexpression of eIF5A restored cell proliferation and invasion after circ-EPB41 silencing, suggesting that circ-EPB41 expression predicts unfavorable prognoses in NSCLC by regulating the miR-486-3p/eIF5A axis.

Previous studies have also found that eIF5A expression can influence tumor progression by regulating tumor stem cell differentiation [20]. CSCs are tumor cells with principal characteristics of clonal tumor initiation capacity, self-renewal and clonal long-term repopulation potential. In general, stem cell marker expression, such as SOX2, OCT-4, Nanog and CD133, with transcriptional signatures particular to CSCs, correlate functionally with aggressive behavior and are closely predictive of patient overall survival. Clinical data indicate that CSCs may be indispensable therapy targets [39, 40]. In the current investigation, we discovered that downregulation of circ-EPB41 suppressed stemness of NSCLC in in vivo and in vitro experiments. Downregulation of miR-486-3p or overexpression of eIF5A restored CSC differentiation after circ-EPB41 silencing, which suggested that circ-EPB41 expression predicts unfavorable prognoses in NSCLC by regulating miR-486-3p/eIF5A axis-mediated stemness. However, the specific regulatory mechanism controlling stemness in NSCLC needs further study.

## Conclusion

In summary, the present study demonstrated that circ-EPB41 was upregulated in NSCLC tissues compared with normal lung tissue, which was related to high proliferation and NSCLC cell invasive activity through sponging of miR-486-3p. Moreover, circ-EPB41 sponged miR-486–3 to promote stemness in NSCLC by enhancing eIF5A expression. Therefore, circ-EPB41 represents as a prognostic marker and a target for NSCLC.

## Supplementary Information

**Additional file 1**: High-throughput sequencing detection show the different expression of circRNA from three NSCLC and three adjacent normal tissue samples.

**Additional file 2**: Bioinformatics analysis prediction the relationship among miR-587, miR-486-3p and miR-595 to circ-EPB41.

**Additional file 3**: High-throughput sequencing for miRNA expression analysis in A549 cells between NC and sh-circEPB41 group.

**Additional file 4**: High-throughput sequencing for mRNA expression analysis in A549 cells between NC and sh-circEPB41 group.

## Data Availability

The data generated or analyzed during this study are included in this article, or if absent are available from the corresponding author upon reasonable request.

## Abbreviations

circRNA: Circular RNA
miR: MicroRNA
shRNA: Short hairpin RNA
NSCLC: Non-small cell lung cancer
RT-qPCR: Quantitative reverse transcription-polymerase chain reaction
FISH: Fluorescence in situ hybridization
eIF5A: Eukaryotic translation initiation factor 5A
CSCs: Cancer stem cell
CCK8: Cell Counting Kit-8
EdU: 5-Ethynyl-2ʹ-deoxyuridine
LC: Lung cancer
NC: Negative control
