# Supplementary figures and images for: Circular RNA EPB41 expression predicts unfavorable prognoses in NSCLC by regulating miR-486-3p/eIF5A axis-mediated stemness
Source: Cancer Cell Int. 2022 Jun 20;22:219. doi: 10.1186/s12935-022-02618-7 (PMC9210757; doi:10.1186/s12935-022-02618-7)

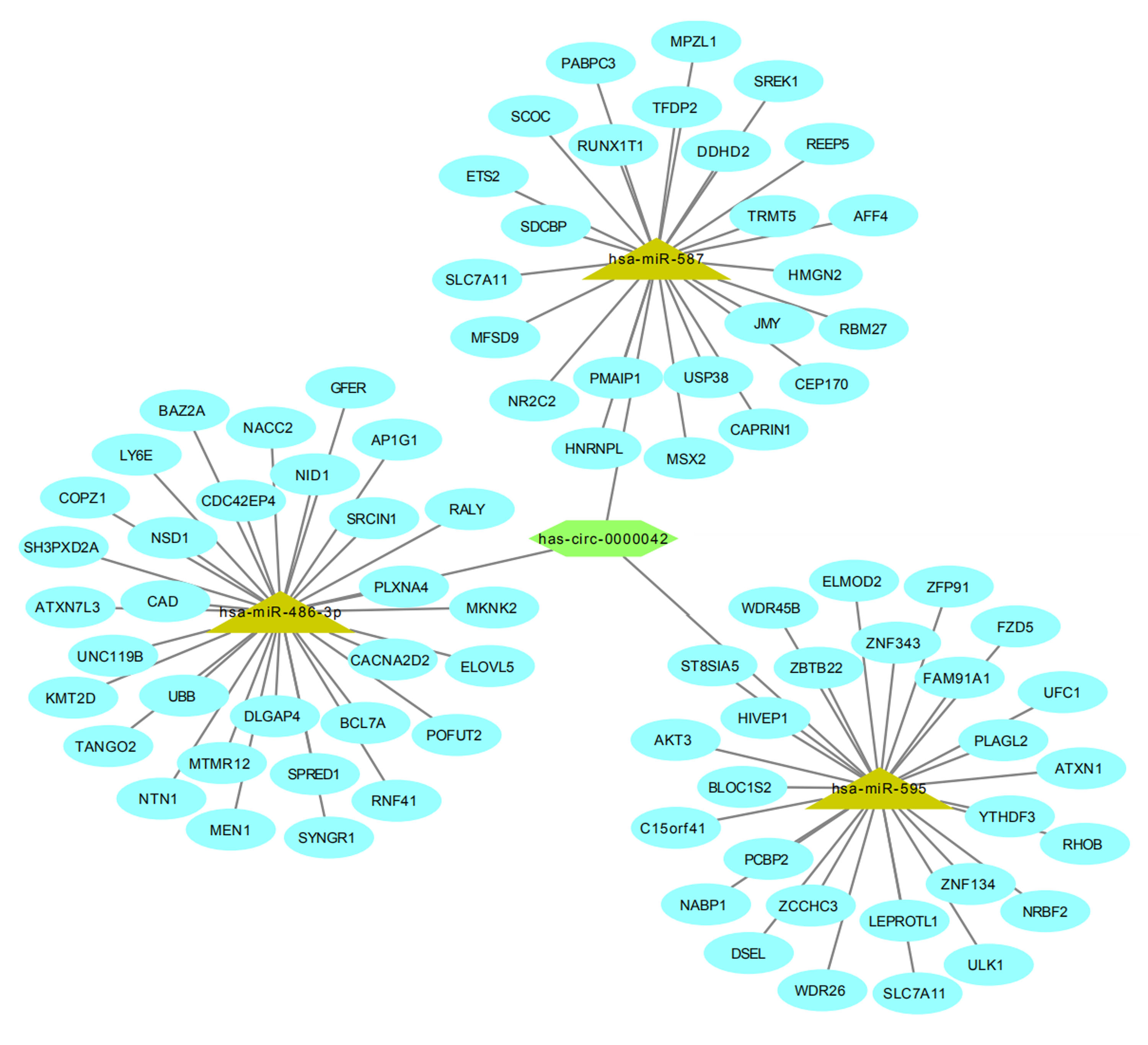

Supplement: Supplementary file 2 — Additional file 2: Bioinformatics analysis prediction the relationship among miR-587, miR-486-3p and miR-595 to circ-EPB41. [file 12935_2022_2618_MOESM2_ESM.doc]
